# Equivalent dose methodology for activity-based comparisons of protease performance during byproduct protein hydrolysis

**DOI:** 10.1016/j.fochx.2025.102596

**Published:** 2025-05-28

**Authors:** Silvana Valdivia, María J. Camus, Tamara Solis, Suleivys Nuñez, Pedro Valencia

**Affiliations:** a Programa de Doctorado en Biotecnología, Pontificia Universidad Católica de Valparaíso/Universidad Técnica Federico Santa María, Valparaíso 2390123, Chile; b Departamento de Ingeniería Química y Ambiental, Universidad Técnica Federico Santa María, Valparaíso 2390123, Chile; c Escuela de Ingeniería Química, Pontificia Universidad Católica de Valparaíso, Valparaíso 2362854, Chile; d Escuela de Alimentos, Pontificia Universidad Católica de Valparaíso, Valparaíso 2360100, Chile

**Keywords:** Protein hydrolysis, Nitrogen extraction, Equivalent doses, Protease performance, Cost-efficiency

## Abstract

An experimental methodology to compare the hydrolysis performance of different proteases based on equivalent doses was proposed and validated. The specific activities were quantified via nonlinear fitting of the logarithmic equation to the reaction progress plot. The obtained kinetic constants *a* were plotted against the protease dose to calculate the specific activities from the slope, resulting in 4214, 1127, 10,277 and 3139 U/g for Alcalase, Flavourzyme, Neutrase and Protamex, respectively. The hydrolysis performances were evaluated using equivalent doses of proteases during the hydrolysis of 50 %(*w*/w) salmon frame at 50 °C and pH 7.5. Higher degrees of hydrolysis were obtained with Flavourzyme (177 mM and 12 %), whereas greater protein recovery was obtained with Alcalase (55 %). Alcalase was the most cost-efficient protease with 4.3 kg of extracted protein per USD. The presented strategy is an affordable methodology that can be applied to different byproduct-protease systems.

## Highlights

• An unbiased method for non-linear activity quantification was validated.
• The proteases' performances were evaluated at equivalent proteases doses.
• Alcalase was the most cost-efficient protease.

## 1 Introduction

The enzymatic hydrolysis of proteins is an attractive technology for the valorization of food byproducts. This technique surpasses other technologies based on thermophysical treatments in two main aspects: the conservation of nutritional value and the production of functional and bioactive peptides (Egerton et al., 2020; Harnedy & FitzGerald, 2012; Wei et al., 2021). In general, the performance of a reaction depends on several main factors, such as the proteolytic susceptibility of proteins, the protease efficiency, and the temperature and pH of the reaction mixture (Valencia et al., 2019). The type of protease and specificity significantly affect the size of the peptides and, consequently, the functional properties of the hydrolysate (Liceaga & Hall, 2019). The available commercial proteases are of microbial origin because of their high efficiency and cost-effectiveness (Kristinsson & Rasco, 2000b; Razzaq et al., 2019). For this reason, the characterization of proteases is a major concern when they are applied for the hydrolysis of byproduct proteins. Unfortunately, the available proteases have been characterized using different activity assays, which impedes rational design of the dose based on the nominal activity reported by the manufacturer. For example, Alcalase and Flavourzyme, both produced by Novozymes, are characterized using Anson units (AUs) and leucine aminopeptidase units (LAPUs), respectively. While the AU is measured by the release of tyrosine during the hydrolysis of hemoglobin quantified via a colorimetric assay using Folin reagent (Akpinar & Penner, 2002), the LAPU is measured by the release of *p*-nitroanilide during the hydrolysis of a peptide-*p*-nitroanilide derivative as directly quantified via photometry (Merz et al., 2015). The activity units are shown in Table 1.

**Table 1:** List of proteolytic activity units of commercial proteases.

Units name (abrev.)	Substrate in assay	Definition of activity	Ref.
Anson units (AU)	Hemoglobin	Amount of extract that produces color equivalent to 1.0 μmole of tyrosine per min at pH 7.5 at 37 °C with Folin-Ciocalteu reagent	(Anson, 1938)
Aminopeptidase units (APU)	Synthetic peptide-*p*-nitroanilide	Amount of extract that produces 1.0 μmole of *p*-nitroanilide per min under assay conditions	(DelMar et al., 1979; Falkenberg et al., 2022; Merz et al., 2015)
Bacterial protease units (BPU)	Casein	Amount of extract that produces color equivalent to 1.0 μmole of tyrosine per min at pH 7.5 at 37 °C with Folin-Ciocalteu reagent	(Silva et al., 2006)
Alkaline Delft units (ADU)	Casein	Amount of extract which after 40 min of incubation at 40 °C gives an absorbance difference of 0.022 at 275 nm	(Teplyakov et al., 1992)
Azocasein units (AzU)	Azocasein	Amount of extract that produces an increase of 0.010 absorbance units per minute under assay conditions.	(Brock et al., 1982; Falkenberg et al., 2022)

Owing to these differences in nominal activity, studies usually compare the performance of different proteases based on mass-equivalent concentrations. An improved approach was proposed by Kristinsson and Rasco (Kristinsson & Rasco, 2000b), where the proteases were characterized using a common activity assay to quantify the specific activity. The protease performance was subsequently tested using equivalent doses based on the quantified activity (Kristinsson & Rasco, 2000b). Despite this improvement, catalytic characterization remains a drawback because of the lack of linearity in the progression curve.

Since the publication of Michaelis and Menten, the study of enzyme behavior by measuring initial rates has been much simpler. The effects of product accumulation and substrate consumption are avoided by this method. Nevertheless, after the characterization of the progress curve, the problem of determining a straight line to estimate the initial rate persists (Cornish-Bowden, 2012). This inherent difficulty is significantly increased in the proteolytic reaction. The enzymatic hydrolysis of proteins is characterized by a very rapid initial phase followed by a dramatic and progressive decrease in the reaction rate (Valencia et al., 2014; Valencia et al., 2019). This curvature is observed even at very short assay times and is explained by strong product inhibition (Valencia et al., 2014). Thus, it does not appear possible to characterize the initial rate of the enzymatic hydrolysis of proteins through a straight-line estimation. As mentioned by Cornish-Bowden (Cornish-Bowden, 2012), the estimation of the initial slope of a curve is subjective and liable to be biased. Several solutions have been proposed to avoid this problem. In 1982, Boeker suggested plotting the instantaneous reaction rate against the product formed (Δ*P*/*t* versus *P*) as a simple and less judgmental alternative to tangent analysis (Boeker, 1982). Unfortunately, less efficient methods have become more popular than this proposal (Cornish-Bowden, 2012). Duggleby's approach recommended the use of numerical integration coupled with nonlinear regression to fit the model to the time courses and estimate the kinetic parameters (Duggleby, 2001). A similar approach was proposed by Tang and Leyh (Tang & Leyh, 2010), where progress curves were transformed into the equivalent of thousands of initial rate and substrate concentration measurements. In addition, the removal of the product by the purified enzymes that drove the reaction forward allowed the influence of product inhibition to be avoided (Tang & Leyh, 2010).

In the present study, a new methodology for the quantification of the initial reaction rate, characterization of the specific activity, and use of equivalent doses of proteases to test their performance during the hydrolysis of byproduct proteins was evaluated. The first stage consisted of activity experiments involving activity assays designed to avoid the subjective estimation of a straight line. Instead, the nonlinear logarithmic equation was used to fit the progress curve during the hydrolysis of salmon-frame proteins by different commercial proteases. The initial rate was obtained from parameter *a* and correlated with the protease concentration. The second stage consisted of hydrolysis experiments where equivalent protease doses were calculated and used to evaluate the reaction performance.

The aim of this study was to evaluate this new methodological approach as a convenient tool for the characterization of protease activity and for the comparison of their catalytic performance under activity-equivalent doses.

## 2 Materials and methods

### 2.1 Reagents and materials

Salmon frames (SFs) were kindly donated by the Fiordo-Austral Group located in southern Chile. Frozen SFs were delivered overnight and processed immediately upon arrival. The nitrogen content in the salmon frames was 2.51 ± 0.06 % *w*/w, which is equivalent to 15.71 ± 0.38 % w/w protein (factor of 6.25; *n* = 4). Proteases were obtained from commercial preparations from Novozymes (Bagsvaerd, Denmark), including Alcalase 2.5 L, a subtilisin with 2.5 AU/g, Flavourzyme 1000 L, an exopeptidase with 1100 LAPU/g, Neutrase 5.0 BG, a neutral metalloendoprotease with 5 AU/g, and Protamex, a blend of neutral metalloendoprotease and subtilisin with 1.5 AU/g. Analytical-grade reagents were used in all the experiments.

### 2.2 Processing of salmon frames and characterization of the hydrolytic activity of proteases

Semifrozen SFs were ground in a Talsa PSV C15 cutter (Valencia, Spain) and then in an RCA RH-950 INOX meat grinder (Santiago, Chile). The ground SFs were stored in 1 kg bags and frozen until use. The hydrolytic activity of proteases was determined during the hydrolysis of 50 g of an SF-water mixture containing 1 % (*w*/w) SF proteins at 50 °C and pH 7.5. The required masses of SF (3.18 g) and water (46.82 g) were weighed and tempered in a water-bath at 50 °C for approximately 5 min. The pH of the reaction mixture was initially about 6.5 and was adjusted to 7.5 by the addition of NaOH 0.05 N. Once the temperature and pH were set, the reaction was started by the addition of protease at concentrations ranging between 0.01 and 0.2 g/L. The pH of the reaction mixture was controlled using the pH-stat technique via the addition of 0.05 N NaOH to a Mettler-Toledo G20 autotitrator which registered the NaOH volume added during the assay. An autolysis experiment was carried out as a control under the same conditions previously described but without the addition of protease. The concentration of α-amino groups (α-NH) released during the hydrolysis reaction was calculated from the titration according to previous studies (Kristinsson and Rasco, 2000a, Kristinsson and Rasco, 2000b; Valencia et al., 2014). Each hydrolysis experiment consisted of two replicates. The logarithmic equation (Eq. (1)) was fitted to the reaction progress plot according to previous studies (Qi & He, 2006; Valencia et al., 2016; Valencia et al., 2019). The data collected from each hydrolysis experiment was used to plot the released α-NH concentration against the reaction time and were used to estimate the parameters *a* and *b* from Eq. 1:

(1) $$P=\left(1/b\right)\;\ln\left(\mathrm{abt}+1\right)$$

where *P* is the released α-NH concentration, *t* is the reaction time, and *a* and *b* are kinetic parameters. The kinetic constant *a* corresponding to the initial reaction rate was plotted against the protease concentration. The slope of this plot corresponds to the specific activity of each protease. One unit of hydrolytic activity (U) was defined as the amount of protease that releases 1 μmole of α-NH groups per minute when SFs were used as substrates under the assayed conditions. Each commercial protease was characterized to quantify its specific activity.

### 2.3 Protease performance during the hydrolysis of SF proteins

Once the specific activity had been estimated, the cost-efficiency of commercial proteases was compared at equivalent doses. The nominal specific activities and prices for each protease are shown in Table 2. Doses of 2.5, 5.0 and 10.0 AU of Alcalase per kg of SF were chosen for the hydrolysis experiments according to previous studies (Valencia et al., 2021; Valencia et al., 2023). The equivalent doses based on the present activity assay were calculated for each protease.

**Table 2:** Nominal activity and price of each commercial protease.

Protease	Nominal Activity	Cost
		(USD/kg)
Alcalase	2.5 AU/g	17.7
Flavourzyme	1100 LAPU/g	74.4
Neutrase	5.0 AU/g	164.3
Protamex	1.5 AU/g	68.9

The hydrolysis experiments were performed in a batch reactor loaded with 400 g of 1:1 SF/water mixture operated with constant agitation for 1 h at 50 °C and a pH of 7.5, which was controlled using a pH-stat. The SF mass (200 g) and water (200 g) were loaded in the reactor and immediately agitated by an impeller at 320 rpm during 15 min. The pH of the reaction mixture was adjusted by the addition of 2 N NaOH. The reaction was initiated with the addition of the protease, and 0.5 mL samples were withdrawn at different reaction times. Each sample was immediately mixed with an equal volume of 10 % trichloroacetic acid and centrifuged for 10 min at 10,000 ×g. The supernatant was recovered and analyzed for the concentration of α-amino groups (α-NH) released. In addition, after 1 h, the reaction mixture was centrifuged at 10,000 ×*g* for 10 min to quantify the soluble phase, nitrogen recovery (NR), and degree of hydrolysis (DH). Each hydrolysis experiment consisted of two replicates.

### 2.4 Analysis and statistics

The total nitrogen contained in the SFs and the soluble phase was quantified via the Kjeldahl method. The free α-NH was quantified via the *o*-phthaldialdehyde (OPA) method using serine as a standard (Nielsen, Petersen, & Dambmann, 2001). The released α-NH groups were calculated by subtracting the initial α-NH group concentration (at time zero) from each value. The reaction mixture was sieved through four layers of gauze to retain the bones, and the fluid phase was centrifuged at 10,000 ×*g* for 10 min to separate the oil, soluble phase, and insoluble phase (pellet). Each phase was weighed.

Nitrogen recovery (NR) was defined as the nitrogen transferred from SFs to the soluble phase and was calculated from the ratio between the total nitrogen in the soluble phase and the total nitrogen in unhydrolyzed SFs. The degree of hydrolysis (DH) was estimated from the ratio between the released α-NH groups and the total nitrogen in the SFs. All hydrolysis experiments were carried out in duplicate. Each sample was assayed in duplicate via the OPA method.

Data from the experimental results were subjected to analysis of variance (ANOVA) to test for significant differences in the means of treatments. The *F*_*0*_ test statistic for the hypothesis of there being no differences in treatment means was calculated and compared to *F*_α,*a*-1,N-a_, where α is 0.05, *a* is the number of treatments and N is the number of experiments. All pairwise means were compared via Tukey's test to determine which treatment means differed. The difference between the means of two treatments was compared to T_α_ = q_α_(*a,N-a*)$\sqrt{{\mathrm{MS}}_{E}/n}$, where α is 0.05, *a* is the number of treatments, *N* is the number of experiments, *MS*_*E*_ is the mean square of the errors and *n* is the number of replicates.

## 3 Results and discussion

Two main aims were formulated for this study. The specific activity was characterized based on the hydrolysis of SFs as the common substrate for all the proteases and the use of equivalent doses among these proteases to evaluate their hydrolytic performance and cost-efficiency.

### 3.1 Characterization of specific activity

**Fig. 1:**
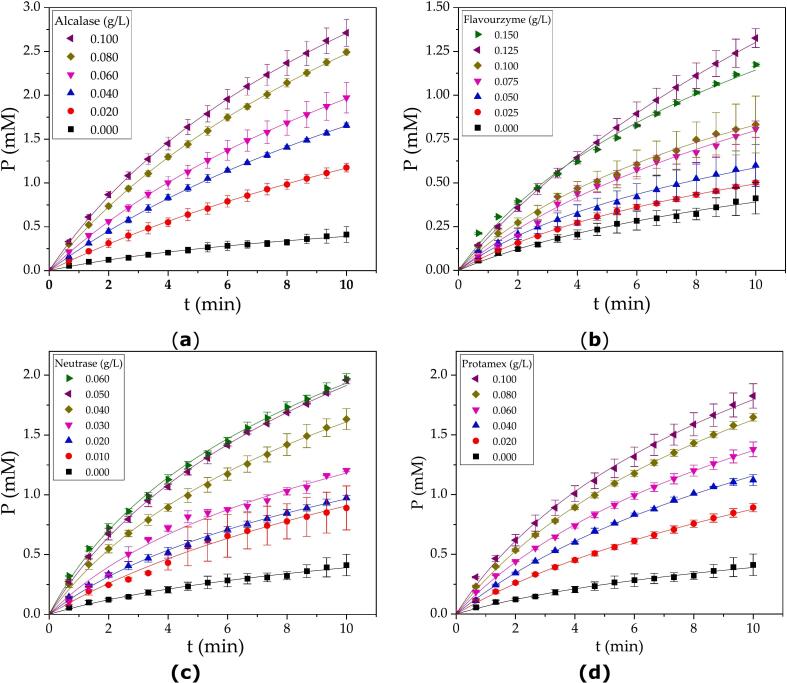
Progression curves during the activity assay for the commercial proteases: (a) Alcalase 2.5 L; (b) Flavourzyme 1000 L; (c) Neutrase 5.0 BG; (d) Protamex, at different protease concentrations. Assay conditions: 50 °C, pH 7.5, and 1 % SF protein. A total of 3600 points were collected in each curve. The plots contain a skipped selection of 1 to 40 points for illustration purposes. The continuous line corresponds to the fitted logarithmic equation (Eq. 1). Each reaction curve consists of an average from two replicates.

The first stage involved activity characterization based on initial reaction rate measurements. The system consisted of a reaction mixture with 1 % SF protein. The reaction progress curves for Alcalase, Flavourzyme, Neutrase and Protamex are plotted in Fig. 1.

**Fig. 2:**
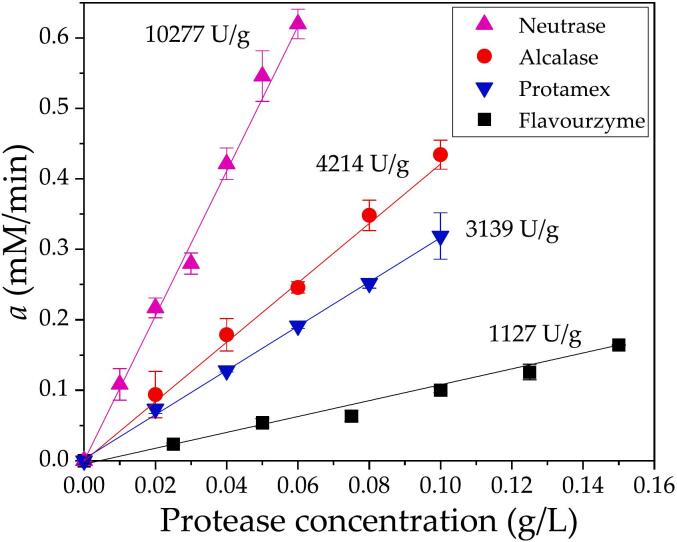
Specific activity of commercial proteases obtained from plotting the kinetic constant *a* against the protease concentration. Each experimental point corresponds to the value of the kinetic constant *a* obtained from each progression curve from Fig. 1. The continuous line corresponds to the fitted linear regression, and the slope represents the specific activity. Assay conditions: 50 °C, pH 7.5, and 1 % SF protein.

The kinetic constants *a* and *b* were estimated from the nonlinear fitting of Eq. (1) to the experimental data. The kinetic constant *a* corresponds to the initial reaction rate of the progression curve, as demonstrated in a previous study (Valencia et al., 2019). The kinetic constant *a* was also demonstrated to be a function of the protease concentration. Although the extent of the reaction was relatively short (10 min), the curvature of the progression plot was observable. The arbitrariness involved in the calculation of a straight line to obtain the initial reaction rate was avoided by using the complete dataset for fitting Eq. (1) via nonlinear regression. The values of the kinetic constant *a* were plotted against the protease concentration in Fig. 2 for each commercial protease, resulting in a linear relationship. The linearity range and slope were different for each commercial protease. Higher protease concentrations were excluded from this correlation because of the lack of linearity (data not shown). A higher slope was obtained for Neutrase, which has higher nominal activity, and the lowest slope was obtained for Flavourzyme. Different values of specific activity were obtained for each commercial protease, as depicted in Fig. 2 and Table 3. There is a correlation between the nominal and measured specific activities of the endopeptidases (Alcalase, Neutrase and Protamex), which can be observed by comparing data from Table 2, Table 3.

**Table 3:** Specific activity and activity costs for commercial proteases used in hydrolysis experiments.

Specific Activity (U/g)		Protease amount for 1 U (g)*10^4^	Activity cost (USD/U)*10^6^
Alcalase 2.5 L	4214	2.37	4.2
Flavourzyme 1000 L	1127	8.87	66.0
Neutrase 5.0 BG	10,277	0.97	16.0
Protamex	3139	3.19	22.0

Notably, the range of protease doses used in the initial rate estimations was between 0.01 and 0.15 g/L (Fig. 2). The maximum dose values were 0.06 g/L for Neutrase, 0.10 g/L for Alcalase and Protamex, and 0.15 g/L for Flavourzyme. These values correspond to 0.6, 1.0 and 1.5 % (*w*/w), respectively, for a mass ratio between protease and SF proteins. These values are important in the discussion of the next stage, where higher doses were used during the hydrolysis performance experiments.

The specific activity determined in the present study and the cost of each commercial protease (obtained from local supplier) can be used to calculate the amount of protease needed to obtain 1 U of activity for the hydrolysis of SF and the cost per activity unit, as presented in Table 3 (Kristinsson & Rasco, 2000b). The lowest cost was obtained for Alcalase, which has the best cost–activity relationship. Flavourzyme is the most expensive protease and has the lowest specific activity. Despite these activity-based criteria are clear and evident, the proper choice of the adequate protease should include the characteristics of the desired hydrolysate, which can be based on the degree of hydrolysis, peptide chain length and functionality. For example, Flavourzyme results in lower bitterness in hydrolysates than endopeptidases (Hou et al., 2011; Liu et al., 2016; Meinlschmidt et al., 2016; Petrova et al., 2018; Seo et al., 2008; Sinthusamran et al., 2020), increases aroma and taste and reduces the texture profile (Feng et al., 2014; Meinlschmidt et al., 2016). Aspects like this should be included in the economic evaluation of the process.

### 3.2 Calculation of equivalent protease doses

Once the specific activity had been obtained, the required equivalent doses based on the nominal activity of Alcalase were calculated (Table 4). This approach considers the proteolytic potential of the different proteases which is far more reliable than comparing them on a weight basis. The calculation was based on Alcalase doses used in previous studies corresponding to 2.5, 5.0 and 10.0 AU per kg SF. Now, these Alcalase doses are equivalent to the actual measured activities corresponding to 4.2, 8.4 and 16.8 U per g SF, respectively. These values are equivalent for all the measured proteases because they were obtained from the same activity assay. Consequently, the amounts of protease that needed to be added to the batch reactor loaded with 400 g of a 1:1 SF/water mixture were calculated and are shown in Table 4. The main significance of these results is the cost associated with the protease loaded in the batch reactor. Neutrase resulted in the lowest amount of protease needed to supply the required activity, followed in order and magnitude by Alcalase (x 2.4), Protamex (x 3.3) and Flavourzyme (x 9.1). Almost 10 times more Flavourzyme is needed for the same activity exerted by Neutrase. This finding can only be observed after applying this methodology, which was previously proposed by Kristinsson and Rasco (2000b) using the Azocoll assay. These authors also observed that comparing proteases with a weight basis gave little insight into the protease efficiency, especially during the hydrolysis of insoluble substrates. In the present study, we have improved the following aspects: i) the activity quantification using the same substrate from the industrial process, and ii) the initial reaction rate estimation based on a non-linear method of fitting that avoids an arbitrary selection of the linear section in a progress reaction plot (*P* vs *t*). The article from Kristinsson and Rasco (2000b) and the present study represent all the literature available regarding the equivalent protease dose for the enzymatic hydrolysis of byproducts proteins. Previously, the use of Azocoll in fish protein hydrolysis was reported by Ferreira and Hultin (1994). The method was proposed as a protease activity method to standardize enzyme activity for fish protein hydrolysis. The use of an insoluble substrate meant a methodological improvement based on reaction conditions more similar to the byproduct characteristics.

**Table 4:** Equivalent doses in quantified units per kg of SF for commercial proteases used in hydrolysis experiments. Reaction conditions: 50 °C, pH 7.5, and 50 % SF.

Dose^1^ (AU/kg SF)	Dose^2^ (U/g SF)	Protease amount needed in batch reactor loaded with 400 g of 1:1 SF/Water mixture (g)			
		Alcalase	Flavourzyme	Neutrase	Protamex
2.5	4.2	0.200	0.748	0.082	0.268
5.0	8.4	0.400	1.495	0.164	0.537
10.0	16.8	0.800	2.99	0.328	1.074

1 Dose in nominal activity units based on Alcalase (from a commercial supplier).

2 Dose in quantified activity units (from this study).

**Fig. 3:**
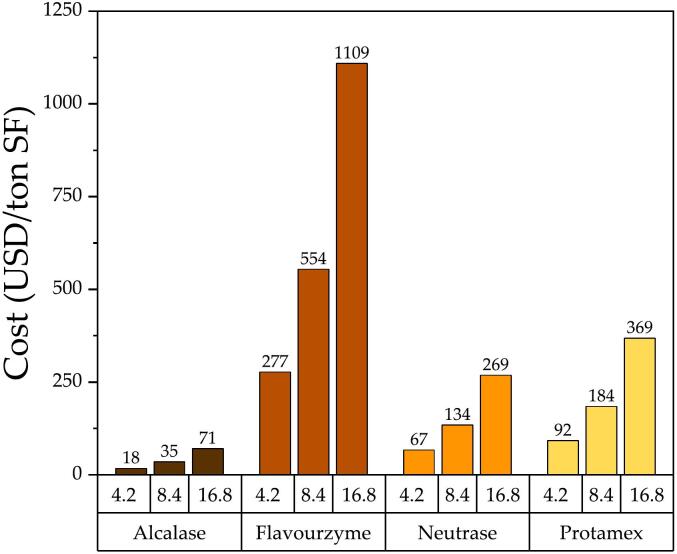
Cost of protease addition for treating 1 ton of salmon frame with different protease doses. Reaction conditions: 50 °C, pH 7.5, and 50 % SF.

The commercial protease Alcalase presented the lowest cost per activity unit, in accordance with the previous study by Kristinsson and Rasco (Kristinsson & Rasco, 2000b). As reported by these authors, Alcalase was more cost-efficient than other commercial and extracted proteases (Kristinsson & Rasco, 2000b). In general, the cost of treating 1 ton of SF for the different doses and proteases was plotted in Fig. 3. Alcalase's costs rise to 18, 35 and 71 USD per ton of SF treated for the corresponding doses of 4.2, 8.4 and 16.8 U/kg, respectively. These values are significantly below the Flavourzyme's costs, which rise to 277, 554 and 1109 USD per ton of SF treated.

### 3.3 Characterization of hydrolytic performance at equivalent protease doses

**Fig. 4:**
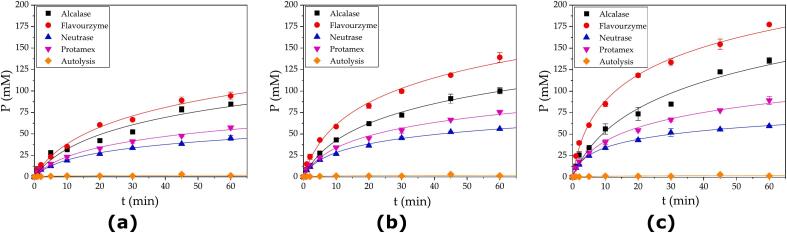
Progression curves during hydrolysis of a 1:1 SF/water mixture by commercial proteases at different doses: (a) 4.2 U/g, (b) 8.4 U/g and (c) 16.8 U/g. Reaction conditions: 50 °C, pH 7.5 and 50 % SF. The continuous line corresponds to the fitted logarithmic equation (Eq. 1).

Hydrolysis experiments were performed with different protease doses in a batch reactor loaded with 200 g of SF and 200 g of water. The proteases were added according to the amounts in Table 4 to initiate the reactions. An autolysis experiment was carried out to determine the effects of endogenous proteases. The hydrolysis curves at three different doses for each commercial protease are shown in Fig. 4. The results indicated that, at the same equivalent dose, the commercial proteases exhibited different hydrolysis performances. The most efficient release of α-NH groups was obtained using Flavourzyme, followed by Alcalase, Protamex and Neutrase. Although, in all cases, an increase in dose caused an increase in the extent of hydrolysis, the magnitude of the change was greater for Flavourzyme, followed by the other proteases in the same order. The observed results are a consequence of the use of the initial reaction rate as a comparison criterion, which is not affected by product inhibition or thermal inactivation of the protease. When the reaction progresses, these factors may significantly affect the reaction performance. It can be inferred that the proteases suffer different degrees of inhibition exerted by the peptides, which was observed in a previous study from our research group (Valencia et al., 2014) and other studies (Apar & Ozbek, 2007; Demirhan et al., 2011; Kristinsson & Rasco, 2000a). We inferred that the different degrees of inhibition observed can be explained by the different peptide compositions generated by each protease and the different affinities between the peptides and the protease. New research is needed to clarify the influence of each factor on the observed hydrolysis efficiency. Experiments consisting in the determination of the initial reaction rate in the presence of protein hydrolysates will allow the characterization of the product inhibition, as performed in our previous study (Valencia et al., 2014).

**Fig. 5:**
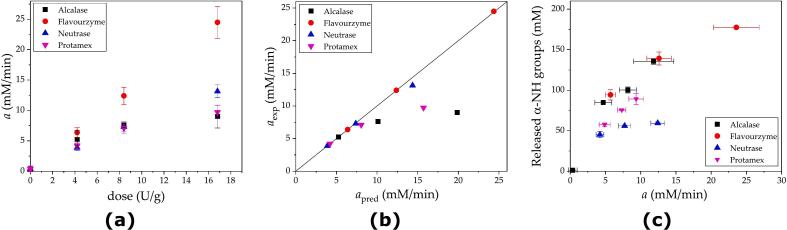
Analyses of hydrolysis performance: (a) kinetic constant *a* plotted against the protease dose, (b) linearity test for the correlation between the experimental *a* values from Fig. 3 and the *a* values estimated via linear prediction, and (c) correlation between the released α-NH groups after 1 h of reaction and the kinetic constants *a* for the different commercial proteases. Reaction conditions: 50 °C, pH 7.5 and 50 % SF.

Several analyses have been developed to compare the quantitative reaction performance of proteases. The first approach was the estimation of the initial rate from each hydrolysis curve via estimation of the kinetic constant *a* from Eq. (1). The values of *a* were correlated with the protease dose, as shown in Fig. 5a, for each commercial protease.

In all cases, the kinetic constant *a* was positively correlated with the protease dose. However, a good linear correlation was observed only for Flavourzyme (R^2^ = 0.99559). Relatively low linear correlations were observed for Neutrase (R^2^ = 0.98244), Alcalase (R^2^ = 0.96799) and Protamex (R^2^ = 0.88081) at relatively high doses, as shown in Fig. 5b. The nonlinear behavior observed in Fig. 5a and b for Alcalase, Neutrase and Protamex is explained by doses that exceed the range of linearity observed in the initial rate estimation assays (Fig. 2) in two- and threefold. On the other hand, the dose of Flavourzyme in the hydrolysis performance experiments was between 2.4 and 9.5 % (*w*/w), whereas the maximum dose used in the initial rate assays was 1.5 % (w/w) for the protease-SF protein ratio. Thus, the maximum dose used in the hydrolysis performance experiments exceeded the range sixfold, but there was still linearity between the initial rate and the dose (Fig. 5b). This very different behavior between Flavourzyme and the other commercial proteases can be attributed to the exoproteolytic nature of this protease compared with the endoproteolytic nature of Alcalase, Neutrase and Protamex (Merz et al., 2015). We infer that endoproteases exert an adsorption dynamic in this heterogeneous system that results in different interfacial enzyme reactions compared to exoproteases, as proposed by Kari et al. (2017).

An additional analysis was conducted to determine the relationship between the reaction product generated after 1 h of reaction and the kinetic constant *a* (initial rate). This analysis was plotted in Fig. 5c and displays the increase in the reaction product when the initial reaction rate was increased. As the initial reaction rate is scarcely affected by product inhibition, in contrast to the advanced reaction rate, we expected to formulate some mechanistic inferences from these observations. The profiles of Alcalase and Flavourzyme were similar in terms of the correlation between the number of released α-NH groups and the kinetic constant *a*. Both proteases produced the highest concentration of released α-NH groups when the initial reaction rate increased. Neutrase showed an almost null increase in the production of α-NH groups at the two higher doses (Fig. 5c). We can infer that Neutrase exhibited a greater degree of inhibition by the released peptides than did Alcalase and Flavourzyme, while Protamex displayed intermediate behavior. Nevertheless, the effects of enzyme saturation cannot be discriminated against from those of product inhibition based on these results. Dedicated experiments are needed to quantify the magnitude of saturation (both the enzyme and substrate) and degree of inhibition. This experimental work is in progress by our research group.

**Fig. 6:**
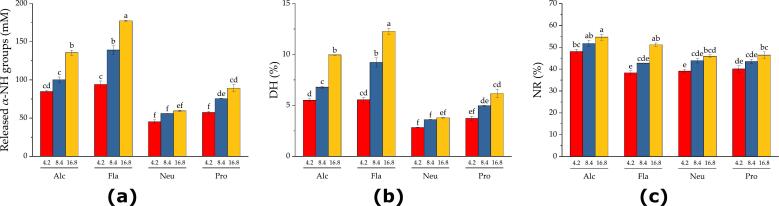
Hydrolysis parameters for the performance of the commercial proteases after 1 h of reaction: (a) released α-NH groups, (b) degree of hydrolysis and (c) nitrogen recovery. Reaction conditions: 50 °C, pH 7.5 and 50 % SF. Different letters indicate significant differences.

The second approach involves evaluating protease performance by calculating different process parameters, such as the concentration of released α-NH, the degree of hydrolysis (DH) and the percentage of nitrogen recovery in the soluble phase (NR) after 1 h of reaction. The data are plotted in Fig. 6.

**Fig. 7:**
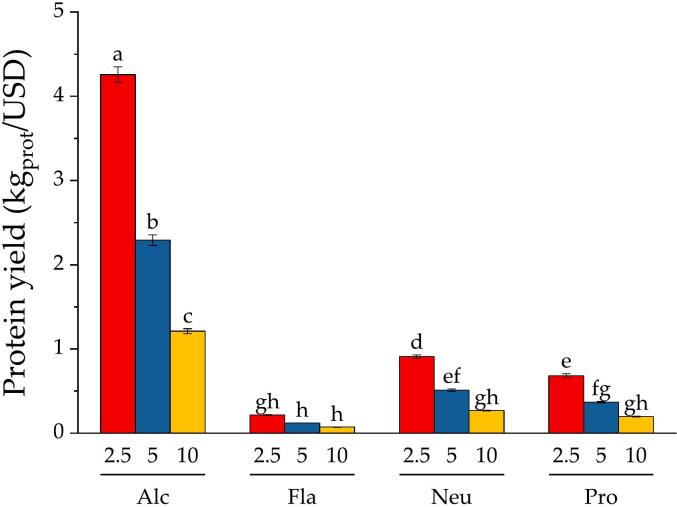
Protein mass obtained per expense dollar in protease cost for the different proteases and doses. Reaction conditions: 50 °C, pH 7.5, and 50 % SF. Different letters indicate significant differences.

The highest concentrations of released α-NH groups (Fig. 6a) and DH (Fig. 6b) were obtained with Flavourzyme. Considering that it is exoprotease, it would be expected to produce shorter peptides and more amino acids compared to endoproteases. Thus, the composition of the hydrolysate is also expected to differ among commercial proteases. The main consequence of these different catalytic actions is observed in the functionality of each hydrolysate. As proteases hydrolyze insoluble SF proteins, soluble peptides and water are released into the aqueous phase, as observed in previous studies (Valencia et al., 2021; Valencia et al., 2023). The transfer of protein from the byproduct to the soluble phase was quantified by nitrogen recovery (NR), which is equivalent to protein recovery. These data are plotted in Fig. 6c. The highest NR content was obtained by Alcalase, followed by Flavourzyme, Protamex and Neutrase. The results for Alcalase were significantly different from those for the other commercial proteases. The NR values ranged from 48 to 55 % for Alcalase, 38 to 51 % for Flavourzyme, 39 to 46 % for Neutrase, and 40 to 46 % for Protamex (Fig. 6c). These results agree with those obtained in our previous studies (Valencia et al., 2021; Valencia et al., 2023). Notably, the concentration of released α-NH groups does not correlate with the NR concentration among commercial proteases. Although Flavourzyme produced the highest concentrations of released α-NH groups, this protease did not produce the highest NR. Despite significant differences being obtained for the α-NH groups among the different proteases (45 to 177 mM), the NR fell within a narrow range for all the proteases (38 and 55 %). NR is a major concern in the production of soluble peptides from protein byproducts because the solubility and peptide composition are the main quality parameters for the commercialization of protein hydrolysates (Liaset et al., 2002; Liaset & Espe, 2008; Valencia et al., 2023). Thus, the economic value of the NR can be evaluated by calculating the amount of protein obtained per expense cost of the protease. The amount of protein hydrolysate per dollar expense for the different protease doses is plotted in Fig. 7.

Enormous differences between Alcalase and the other commercial proteases were observed. Alcalase yielded values between 1.2 and 4.2 kg/USD, whereas the other proteases yielded values between 0.07 and 0.91 kg/USD. These results agree with a previous study by Kristinsson and Rasco (2000b), where Alcalase 2.4 L was the most cost-efficient among commercial proteases and a visceral serine protease. In the present study, Alcalase 2.5 L exerted a significantly higher effect than Flavourzyme, Neutrase and Protamex. The high ratio between the amount of protein hydrolysate produced and the protease cost explains the observed results. Although Flavourzyme generated a relatively high concentration of released α-NH groups and DH, it did not yield a relatively high NR. This result combined with a higher protease and activity cost (Table 2, Table 3) resulted in the lowest protein hydrolysate yield (Fig. 7). With all this evidence we can conclude that Alcalase is the most cost-efficient protease to hydrolyze salmon frames' proteins. Although Alcalase has also resulted in higher efficiency in other studies, we cannot extrapolate these findings to a general conclusion that Alcalase is the best protease to hydrolyze byproduct's proteins. Some proteins such as collagen have a very special composition. The antecedents suggest different combinations of proteases to hydrolyzed collagen (Abdollahi et al., 2018). The high content of glycine and proline in collagen can cause low hydrolysis efficiency by Alcalase, which exhibits high specificity for cleaving peptide bonds formed by aromatic amino acids (Tacias-Pascacio et al., 2020). Alcalase also produces peptides with higher bitterness than other prtoeases. The relative bitterness obtained with Alcalase was 5.5, whereas it was 4.1 for Flavourzyme and 3.4 for a mixture of bromelain and papain, according to Petrova et al. (Petrova et al., 2018). Mixtures of *endo*- and exoproteases are recommended to overcome the bitterness of fish protein hydrolysates because exopeptidases work by splitting off hydrophobic amino acids from bitter peptides, thus decreasing their bitterness (Kristinsson & Rasco, 2000a). The search for an appropriate mixture of proteases can now be pursued using the present methodology as the basis for a technical-economical evaluation based on equivalent protease doses.

The usefulness of the presented methodology requires the evaluation of two important issues: i) the linearity range for the specific activity of proteases, and ii) the evaluation of the proteases' performance. These requirements are necessary to make this methodological tool work properly. We have observed that even at equivalent doses proteases may exhibit different hydrolysis performance in terms of degree of hydrolysis and protein extraction. Once completed the equivalent dose evaluation, the costs can be calculated and further proceed with the evaluation of other technical aspects of the process, such as the quality of the hydrolysates functional properties.

## 4 Conclusion

A methodology for the evaluation and comparison of protease performance during the hydrolysis of byproduct proteins was proposed herein. The equivalent protease dose methodology achieved two main milestones: i) the avoidance of an arbitrary choice of linearity during the estimation of the initial reaction rate for the quantification of the specific activity and ii) an equivalent comparison of the cost-efficiency of the proteases for an economical evaluation of the process. This methodology can be readily applied to different combinations of byproducts and proteases, thus allowing both mechanistic inferences to be verified by subsequent research and a clear estimation of the costs and yields. An affordable tool for the technical and economical evaluation of the protein valorization of byproducts is currently available.

## CRediT authorship contribution statement

**Silvana Valdivia:** Writing – original draft, Visualization, Validation, Methodology, Investigation, Formal analysis, Conceptualization. **María J. Camus:** Visualization, Validation, Methodology, Investigation, Formal analysis, Conceptualization. **Tamara Solis:** Visualization, Validation, Methodology, Investigation, Formal analysis. **Suleivys Nuñez:** Writing – review & editing, Writing – original draft, Visualization, Validation, Supervision, Project administration, Methodology, Investigation, Formal analysis, Conceptualization. **Pedro Valencia:** Writing – review & editing, Writing – original draft, Visualization, Validation, Supervision, Resources, Project administration, Methodology, Investigation, Funding acquisition, Formal analysis, Conceptualization.

## Funding

This research was funded by the Chilean National Agency of Research and Development (ANID) through the following projects: FONDECYT Regular [grant number 1231328], FONDECYT Iniciación [grant number 11250136] and ANID PIA [grant number ACT192162].

## Declaration of competing interest

The authors declare that they have no known competing financial interests or personal relationships that could have appeared to influence the work reported in this paper.

## Data Availability

No data was used for the research described in the article.
